# Gender bias in the diagnosis of Alzheimer's Disease

**DOI:** 10.21203/rs.3.rs-10738736/v1

**Published:** 2026-09-11

**Authors:** Susanne Neufang, Atae Akhrif, Oya D Beyan, Neuroimaging Initiative

**Affiliations:** University of Cologne, University Hospital Cologne; University of Cologne, University Hospital Cologne; Alzheimer’s Disease Neuroimaging Initiative

**Keywords:** Bioinformatics, Gender Bias, Bias Mitigation Algorithms, Alzheimer’s disease, XAI

## Abstract

Fairness in clinical machine-learning (ML) remains a critical challenge, particularly in neurodegenerative disease where demographic factors may confound disease-related patterns. We developed and evaluated bias-aware ML models for Alzheimer’s disease (AD) classification using data from the Alzheimer’s Disease Neuroimaging Initiative (ADNI), comprising 757 individuals with mild cognitive impairment (MCI) and AD. Four supervised learning algorithms (logistic regression, support vector machine, random forest, and XGBoost) were trained using demographic, genetic, neuropsychological, and MRI-derived features. Sex was treated as a protected attribute, and bias mitigation was implemented using pre-processing, in-processing, and post-processing methods within the IBM AI Fairness 360 framework.

Bias mitigation reduced disparities in predictions, with disparate impact decreasing from 1.167 to 0.823. The logistic regression model with Disparate Impact Remover (LR/DIR) improved performance while maintaining fairness, whereas adversarial debiasing achieved the highest balanced accuracy (88.91%). Explainability analyses in the LR/DIR model using LIME showed altered feature attribution patterns with increased convergence between sexes and stronger contribution of clinical and gender-related variables after debiasing. SHAP analysis in the adversarial debiasing model confirmed that Mini-Mental State Examination score, functional status, age, and medial temporal lobe atrophy were the strongest predictors of AD.

These findings demonstrate that fairness-aware ML can reduce sex-related bias in AD classification without loss of performance and improve interpretability through more balanced representation of disease determinants.

## Introduction

Alzheimer’s Disease (AD) is a progressive neurodegenerative disorder that leads to memory loss, cognitive decline ^1^, neural alterations and behavioral changes ^2,3^. Women have a higher lifetime risk of developing AD than men, partly because they have a longer life expectancy (approximately 20% versus 10% risk at age 45). They also exhibit distinct disease trajectories, with women progressing nearly twice as fast as men after diagnosis. ^4^. Women have a lifelong advantage in verbal memory ^5^ and often show better cognitive performance than men despite similar levels of hippocampal neurodegeneration ^6^. This may delay diagnosis of mild cognitive impairment (MCI), leading to faster apparent decline once AD is identified ^7^. This pattern has been observed across several neuroimaging biomarkers, including hippocampal atrophy, amyloid PET, and reduced brain glucose metabolism ^6,8,9^. Educational and social factors—including caregiving burden, stress, and social networks—also influence disease presentation. Because women are more often primary caregivers, chronic stress may contribute to depression, sleep problems, and subjective cognitive complaints, which can worsen memory and word-finding difficulties ^10^. Larger, more active social networks and frequent verbal interaction may delay cognitive decline ^11^. In contrast, social isolation—more common in some older women—is linked to poorer memory and language performance ^12^. Marital status also influences dementia risk, with unmarried, widowed, or lifelong single individuals showing higher risk than married individuals ^13^ as marriage may also provide cognitive stimulation and earlier detection of health issues ^14^. The APOE gene is a major genetic risk factor for AD, with the ε4 allele strongly increasing risk of late-onset AD ^15^. While both sexes are affected, women carrying ε4 may show faster cognitive decline and earlier onset than men ^16^. This difference may be partly explained by estrogen’s neuroprotective effects, which decline after menopause and may worsen APOE ε4–related risk ^17^. As a result, postmenopausal women—especially those with the ε4/ε4 genotype—may be at particularly high risk for AD ^18^. Finally, comorbid depression may contribute to sex differences in AD detection. Midlife depression increases AD risk by up to 70% and is more common in women, particularly during the menopausal transition ^19,20^. This may obscure early AD, as depressive symptoms and subjective cognitive decline in women can be misattributed to psychiatric causes. Neuroimaging studies also suggest greater hippocampal and brain volume loss in women with MCI, with depression-related atrophy observed mainly in women ^21,22^ (for a summary see Table 1).

AI models achieve high accuracy in AD diagnosis from MRI and PET, especially for AD vs. controls, but perform worse for MCI due to overlapping pathology ^23,24^. Multimodal approaches improve performance, yet clinical translation is limited by poor generalizability, class imbalance, and limited interpretability ^25,26^. Evidence also suggests sex-related differences in performance, with lower sensitivity for early-stage AD in women ^27^. Despite increasing awareness of sex differences in AD, only a handful of AI studies have explicitly evaluated model fairness across sex, and even fewer have implemented formal bias-mitigation techniques ^28^. Hence, fairness-aware AI for AD diagnosis remains an emerging research area.

AI fairness can be assessed at both the sample and model levels. Sample bias is commonly quantified using the standardized mean difference (MD), which measures imbalance in baseline characteristics between demographic groups. Model fairness is evaluated using metrics such as disparate impact (DI), which compares positive prediction rates, and equalized odds difference (EOD), which assesses differences in sensitivity and specificity across groups. In the clinical context, EOD is considered particularly relevant because it accounts for true disease status, providing a clinically meaningful measure of sex-related differences in diagnostic performance. Methods to mitigate existing bias address bias at three stages: pre-processing (data adjustment), in-processing (algorithm modification), and post-processing (prediction adjustment). Whereas pre-processing (e.g., Disparate Impact Remover (DIR), Reweighing) and post-processing methods (e.g., Reject Option Classification) are applied to or on top of a base classifier, in-processing methods (e.g., Adversarial Debiasing, Prejudice Remover) modify or replace the learning algorithm and are trained directly on the training data. The mitigation methods correct bias in different ways. DIR transforms feature distributions to reduce their correlation with the protected attribute while preserving as much predictive information as possible. Reweighing assigns different sample weights to demographic groups and class labels the training data. Reject Option Classification adjusts decision boundaries after model training by favoring the unprivileged group in cases of prediction uncertainty. The aim in these methods is to increase the influence of gender-related features. Among in-processing methods, Adversarial Debiasing jointly trains a classifier and an adversarial network that predicts the protected attribute, encouraging the classifier to learn representations that are predictive of the target outcome but uninformative of the protected attribute (in our case sex-unrelated). Prejudice Remover incorporates a fairness regularization term into the loss function to penalize dependence between predictions and the protected attribute during training. Bias mitigation often is combined with model interpretation techniques (explainable artificial intelligence (XAI)). Among the most widely used post-hoc explanation techniques are SHapley Additive exPlanations (SHAP) and Local Interpretable Model-agnostic Explanations (LIME). SHAP computes feature attributions by estimating each feature’s contribution to the prediction across all possible feature combinations. It assigns each feature a Shapley value, representing its average marginal contribution to the model output. LIME, in contrast, explains individual predictions by approximating the complex model locally with an interpretable surrogate model, typically a sparse linear model. It perturbs the input around a specific instance and fits the surrogate model to the resulting predictions, thereby identifying which features most influence the model’s decision in that local neighborhood.

In this study, we developed debiased models for the diagnosis of AD patients. We hypothesized that ML models trained on data of the Alzheimer's Disease Neuroimaging Initiative (ADNI) data would exhibit gender-related bias in the diagnosis of AD. We expected that applying fairness-aware bias mitigation techniques would reduce these disparities without degrading—and potentially improving—overall classification performance. Therefore, we used five different bias mitigation methods (Reweighing and DIR (pre-processing), Adversarial Debiasing and Prejudice Remover (in-processing), and Reject Option Classification (post-processing) and four base models, representing different methodological families: logistic regression (LR, linear model), support vector classification (SVC, kernel-based method), random forest (RF, bagging ensemble), and XGBoost (gradient boosting ensemble). Finally, we hypothesized that debiasing would shift model reliance toward more clinically relevant and disease-specific features rather than demographic proxies.

## Methods

### Study population

Data used in this study were obtained from the ADNI database (adni.loni.usc.edu). ADNI was launched in 2003 as a public–private partnership led by Michael W. Weiner, MD, with the primary objective of evaluating whether longitudinal magnetic resonance imaging (MRI), positron emission tomography (PET), biological markers, and clinical and neuropsychological assessments can be combined to monitor disease progression in MCI and AD. The clinical coordination center of ADNI established a network of clinical sites and developed a plan for the recruitment and retention of subjects, and furthermore prepared the final clinical protocol and informed consent, which is distributed to the sites for local institutional review board approval^29^.

The cross-sectional study cohort comprised 757 participants (337 women), including 600 individuals with MCI (269 women) and 157 with AD (68 women). Participants were between 55 and 108 years of age (Table 2).

The final dataset contained 129 variables, including the outcome variable (diagnosis), the protected attribute (sex), nine diagnostic variables, eight demographic variables, seven Clinical Dementia Rating (CDR) items, 11 Functional Assessment Questionnaire (FAQ) items, 34 Mini-Mental State Examination (MMSE) items, five APOE genotypes, and 52 MRI-derived brain volume measures. Brain volumes were normalized to intracranial volume to account for individual differences in head size. A complete list of variables is provided in **Supplementary Table s1**.

### Machine learning workflow

Data preprocessing was conducted in accordance with standard prediction modelling practices. Metadata variables, including diagnosis flags and DXMPTR* proxy columns (n = 18), were removed prior to analysis to prevent leakage of outcome-related information. Features exhibiting zero variance or constant values across the cohort were excluded, as they provided no discriminative information for model training. For numerical variables, missing values were imputed using the feature-wise median calculated within the training dataset to preserve robustness to outliers and maintain distributional stability.

Four supervised machine learning algorithms representing complementary methodological families were evaluated: LR, SVM, RF, and XGBoost.

Prior to model training, continuous variables were standardized using StandardScaler, and class imbalance was addressed using Synthetic Minority Oversampling Technique (SMOTE) only in the training data. The dataset was randomly divided into training (70%) and test (30%) sets. Hyperparameter optimization was performed within the training data using five-fold stratified cross-validation (StratifiedKFold) and GridSearchCV.

The final optimized models were:

- Logistic Regression: C = 1.0, L2 regularization, maximum iterations = 1000
- Support Vector Machine: polynomial kernel, C = 0.1, γ = 0.1
- Random Forest: 200 trees, maximum depth = 6, minimum samples per leaf = 5
- XGBoost: 200 trees, maximum depth = 4, learning rate = 0.1, subsample = 0.8

Random seeds were fixed where applicable to ensure reproducibility.

### Bias Detection and Mitigation in Model Development

To mitigate bias in the models, we applied pre-processing (Reweighing and Disparate Impact Remover), in-processing (Adversarial Debiasing, Prejudice Remover), and post-processing (Reject Option Classification) bias mitigation algorithms. All models were trained twice, on the original dataset as well as on the debiased dataset. Protected attribute was sex, with privileged_groups = men and unprivileged_groups=women. Therefore, the bias addressed in the current analyses will refer to sex bias. Fairness was evaluated using outcome metric EOD and DI (Fig. 1). Because adversarial debiasing is sensitive to random initialization, models were trained using seeds 0–99, and the model exhibiting the most favorable combination of predictive performance and fairness metrics was selected. Bias mitigation algorithms were implemented using IBM AI Fairness 360 (https://ai-fairness-360.org/).

Figure 1 depicts the ML workflow, in blue all mitigation-related analysis steps are presented, in green additional XAI steps.

### Local explainability of corrected predictions

To investigate how debiasing altered individual predictions, LIME, were generated for participants who were misclassified before debiasing and correctly classified after debiasing. Local feature importance was computed using explain_instance() with all available features. Analyses focused on participants with AD who were incorrectly classified as MCI before bias mitigation but correctly classified as AD after debiasing. LIME feature weights were extracted before and after debiasing and analysed separately for women and men. One-sample t-tests were performed to determine whether mean feature contributions differed significantly from zero within each subgroup. The applied statistical threshold was p < .05, corrected for multiple comparisons using Bonferroni correction.

### Global explainability of debiased models

Global model behaviour was investigated using SHAP. SHAP summary (beeswarm) plots were generated to visualize the distribution and magnitude of feature contributions across all participants. Features were ranked according to their mean absolute SHAP values, and SHAP summary bar plots were used to quantify overall feature importance. SHAP was selected for the adversarially debiased model because it provides theoretically consistent global feature attributions, enabling evaluation of whether the debiased model continued to rely on disease-relevant predictors rather than the protected attribute or its proxies

All analyses were conducted in Python 3.11. Machine learning models were implemented using scikit-learn and TensorFlow. Bias mitigation was performed using the IBM AI Fairness 360 (AIF360) toolkit, and class imbalance was addressed using the imbalanced-learn library. Explainability analyses were conducted using LIME and SHAP. Data preprocessing and numerical computations were performed using NumPy, pandas, and SciPy. Visualizations were generated using Matplotlib. Interactive development and reproducibility checks were carried out in Jupyter Notebook. Exact package versions are provided in the Supplementary Material to ensure reproducibility (**Table s2**).

## Results

### Quantification of sample bias

The original dataset exhibited an MD of 0.038 and a DI of 1.167. Following debiasing, MD decreased to − 0.036 and DI to 0.823.

### Bias mitigation performance

Baseline model performance ranged from 85.16% to 88.32% bA, with the RF model achieving the highest accuracy. Among the base models, LR demonstrated the lowest bias, with a DI of 0.997 (95% CI, 0.93–1.22), an SPD of − 0.017 (95% CI, − 0.03 to 0.03), and an EOD of − 0.036 (95% CI, − 0.19 to 0.13). Among the pre-processing approaches, the SVM model combined with DIR corrected the largest number of previously misclassified participants. However, its DI exceeded 1, indicating potential overcorrection. The LR model combined with DIR correctly reclassified eight participants while increasing classification accuracy from 85.16% to 88.08%. Fairness metrics were maintained or improved, with DI increasing from 0.997 to 1.02, SPD from − 0.017 to 0.010, and EOD from − 0.036 to − 0.003. Based on the combined evaluation of predictive performance and fairness metrics, the LR/DIR model was selected for subsequent explainability analyses (Table 3). The in-processing Adversarial Debiasing model (seed = 49, selected from a sweep of seeds 0–99) achieved an accuracy of 88.91%, with a DI of 0.997 and an SPD of − 0.001 (Table 3). Based on comparable performance and bias, this model also entered subsequent explainability analysis. For results using class balancing instead of SMOTE see **Table s3**.

### Explainability of the LR/DIR model

The LR/DIR model correctly reclassified eight participants who had been misclassified before debiasing, including three women and five men. LIME explanations were generated separately for AD converters and MCI converters (n = 4 per subgroup) to examine sex-specific feature importance. Before debiasing, the significant features differed between sexes. Men showed significant functional and biological features, whereas women showed only biological features, with no overlap between the two groups. Following debiasing, caregiving skills and left BA36 atrophy were identified as significant features in both sexes, indicating greater convergence in feature importance. Retirement status and APOE ε2/ε3 genotype were additionally identified in both groups, whereas comorbid depression remained significant only in women. The number of gender-related features increased following debiasing in both women and men (Fig. 2).

Figure 2. Results from XAI of the LR/DIR model: significantly important features before and after debiasing. In red letters, features are highlighted, which are associated with gender gap. Blue barplots show male-specific features, red female-specific significant features. APOE: Apolipoprotein E, ethnic category: 1 = Hispanic or latino, 2 = non-hispanic, 3 = unknown [N = 1], CDR: clinical dementia rating, DEMOG: patient demographic information, BA: Brodman area. Significant positive scores deviation from 0 indicate an increase in the predicted probability for AD and vice versa, the larger the magnitude (absolute value) the stronger the influence.

### Explainability of the Adversarial Debiasing model

SHAP analysis identified MMSE total score, FAQ total score, age, and medial temporal lobe volume as the strongest contributors to model predictions. The SHAP beeswarm plot showed that lower MMSE scores and smaller medial temporal lobe volumes were associated with a higher probability of AD classification, whereas higher age and higher FAQ scores were associated with an increased probability of AD (Fig. 3).

Figure 3. Results from XAI of Adversarial Debiasing Model: important features after debiasing. In red letters, features are highlighted, which are associated with gender gap. CDR: Clinical Dementia Rating, MMSE: Mini Mental State Exam, FAQ: Functional Activity Questionnaire

## Discussion

Our findings demonstrate that algorithmic fairness interventions can substantially reduce sex-related disparities in AD classification without compromising diagnostic performance. Both the LR–DIR model and the Adversarial Debiasing model achieved fairness metrics approaching parity while maintaining high accuracy. These findings challenge the commonly assumed trade-off between fairness and predictive performance and support recent recommendations that fairness evaluation should be integrated throughout the development of clinical AI rather than considered only after model development. Recent reviews have similarly concluded that algorithmic bias frequently arises from characteristics of the training data rather than inherent limitations of ML models, making bias mitigation an essential component of trustworthy AI in healthcare ^30-32^.

The present findings are particularly relevant in AD because biological sex influences disease risk, neuropathology, clinical presentation, and cognitive reserve. Women account for nearly two-thirds of individuals living with AD and exhibit differences in amyloid accumulation, tau pathology, hippocampal atrophy, and rates of cognitive decline that cannot be explained solely by their longer life expectancy ^27,33,34^. These biological differences are further complicated by gender-related differences in education, depression, and healthcare utilization, all of which may introduce systematic bias into observational datasets such as ADNI ^35^. Consequently, machine learning algorithms trained on such datasets may inadvertently learn demographic patterns rather than disease-specific biology. Fairness-aware learning therefore represents an important strategy for distinguishing clinically meaningful sex differences from algorithmic bias.

The local explainability analyses provide important insight into how debiasing modified model behaviour. Before bias mitigation, corrected predictions relied on substantially different features in women and men, suggesting that the classifier had learned sex-specific decision pathways. Following debiasing, caregiving ability and left Brodmann area 36 atrophy emerged as significant contributors in both sexes, indicating greater convergence in diagnostic reasoning. Notably, debiasing also increased the contribution of gender-related variables, including caregiving responsibilities and retirement status, while preserving clinically relevant biological features such as APOE genotype and medial temporal lobe atrophy. This pattern suggests that the fairness intervention did not simply reduce reliance on sex-specific decision rules but recalibrated the model to integrate both biological determinants of AD and socially mediated factors that may influence disease presentation and diagnosis ^27,36,37^.

Depression remained influential only among women after debiasing. Rather than representing residual algorithmic bias, this finding is consistent with epidemiological evidence showing that depression is more prevalent in women and is associated with an increased risk of cognitive decline and dementia ^38,39^. More broadly, recent dementia research has emphasized that sex and gender represent distinct but interacting determinants of brain health: sex influences neurobiology, whereas gender shapes education, occupation, caregiving roles, healthcare access, and other life-course exposures that contribute to cognitive reserve and dementia risk ^37,40^. Our findings suggest that fairness-aware ML may facilitate a more balanced representation of these complementary determinants rather than preferentially weighting one dimension over the other.

The global SHAP analysis further supports the biological validity of the debiased models. SHAP was specifically applied to the Adversarial Debiasing model because this in-processing approach learns latent representations that minimize information about the protected attribute while preserving predictive performance ^30,31^. Consequently, unlike the local LIME analyses—which were designed to investigate how individual corrected predictions differed between women and men—SHAP enabled the interpretation of a model whose predictions were expected to rely less on sex-related information and more on disease-relevant features ^32,33^. The SHAP analysis identified MMSE score, FAQ score, age, and medial temporal lobe volume as the strongest determinants of model predictions. These variables are well-established markers of AD severity and progression and are among the most reproducible predictors identified across imaging and multimodal machine learning studies using ADNI and other cohorts ^27,34,35,39^. The absence of sex as a dominant contributor in the global feature rankings further suggests that the adversarial training successfully reduced dependence on the protected attribute while preserving clinically meaningful biological signals. Collectively, these findings indicate that fairness-aware learning redistributed feature importance without fundamentally altering the biological basis of the model, supporting the use of adversarial debiasing to promote equitable and clinically interpretable decision-making.

Our results also demonstrate the complementary value of combining fairness assessment with XAI. Fairness metrics quantify disparities in model outcomes but provide limited insight into the mechanisms responsible for those disparities. Conversely, explainability methods identify influential features but cannot determine whether predictions are equitable across demographic groups. By integrating demographic fairness metrics with local (LIME) and global (SHAP) explanations, our framework provides both quantitative assessment of algorithmic bias and mechanistic understanding of model behaviour. Similar recommendations have recently been proposed for trustworthy clinical AI, where fairness, transparency, and interpretability are increasingly viewed as complementary rather than independent components of responsible AI development ^31,32,41,42^.

Several limitations should be acknowledged. First, ADNI is a highly curated research cohort with relatively high educational attainment, limited ethnic diversity, and standardized imaging protocols, potentially limiting generalizability to routine clinical populations. This limitation has been widely recognized in systematic reviews of machine learning for dementia diagnosis ^43,44^. Second, sex was treated as a binary protected attribute because this information is available in ADNI; consequently, our analyses do not address gender identity or intersectional sources of bias involving race, ethnicity, education, or socioeconomic status. Third, fairness was evaluated using demographic parity and equal opportunity, but no single fairness metric fully captures all dimensions of equitable clinical decision-making ^30^. Finally, although LIME and SHAP provide complementary explanations, both methods rely on methodological assumptions regarding local approximations and feature dependence that should be considered when interpreting feature attribution ^42^.

Future work should validate fairness-aware diagnostic models in independent cohorts such as the Australian Imaging, Biomarkers and Lifestyle Flagship Study of Ageing (AIBL), the National Alzheimer's Coordinating Center (NACC) database, and the Open Access Series of Imaging Studies (OASIS), while evaluating performance prospectively within heterogeneous healthcare systems. Longitudinal studies are also needed to determine whether fairness-aware learning improves prediction of progression from MCI to AD while maintaining equitable performance across demographic groups. Extending fairness analyses beyond sex to include race and ethnicity, socioeconomic status, and intersectional identities will be essential for developing equitable clinical decision-support systems.

In conclusion, fairness-aware ML substantially reduced sex-related disparities in AD classification while maintaining high diagnostic performance. Integrating algorithmic debiasing with complementary explainability approaches revealed that improvements in fairness were accompanied by more consistent feature attribution across women and men. Importantly, debiasing not only attenuated reliance on sex-specific decision patterns but also increased the relative contribution of gender-related variables, including caregiving responsibilities and retirement status, alongside established biological markers of AD. This shift suggests that fairness interventions may promote a more balanced representation of both biological and socially mediated determinants of disease within predictive models, without compromising clinical interpretability. Collectively, these findings support the routine integration of fairness auditing and explainability in clinical AI development and highlight the potential of fairness-aware models to provide more equitable and contextually informed decision support in neurodegenerative disease.

## Supplementary Material

The online version contains supplementary material available

This is a list of supplementary files associated with this preprint. Click to download.

• Supplement.docx

## Figures and Tables

**Figure 1:**
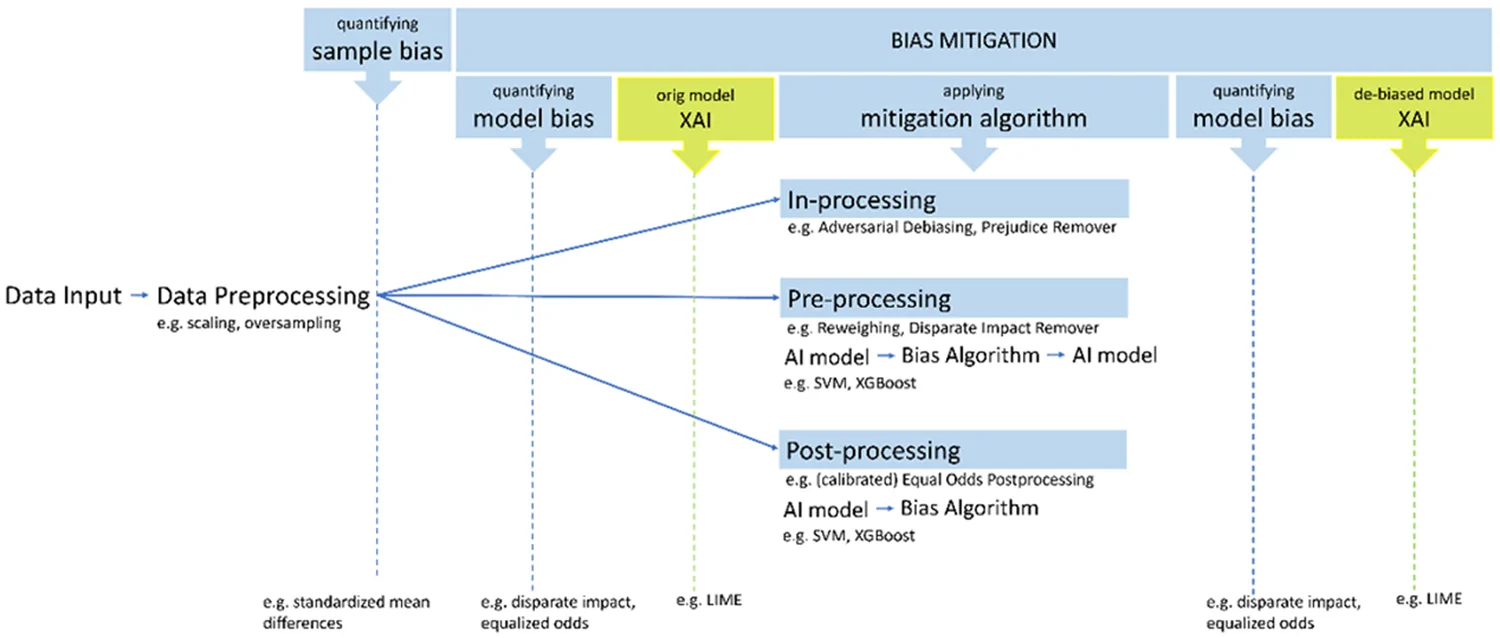
depicts the ML workflow, in blue all mitigation-related analysis steps are presented, in green additional XAI steps.

**Figure 2:**
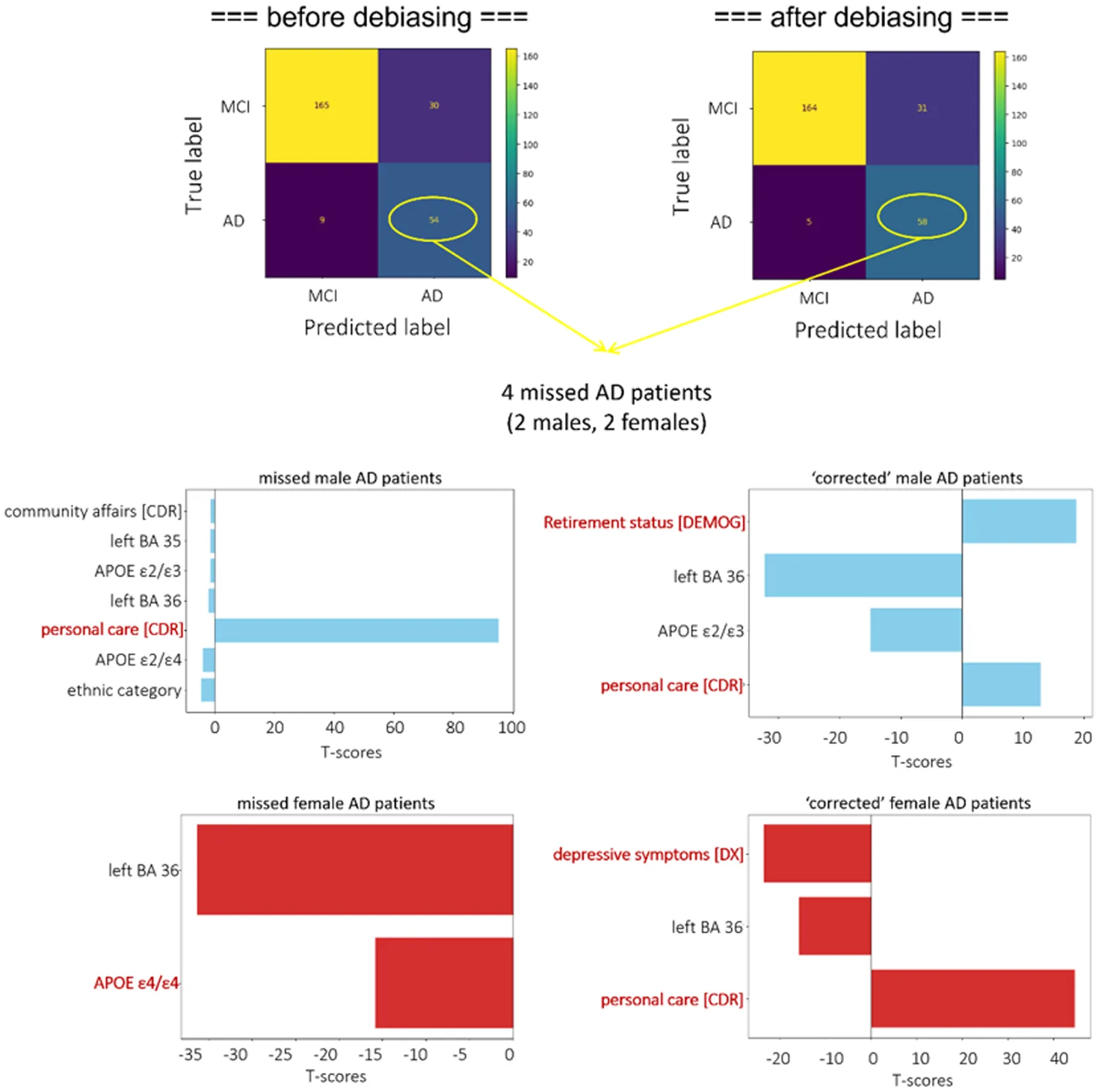
Results from XAI of the LR/DIR model: significantly important features before and after debiasing. In red letters, features are highlighted, which are associated with gender gap. Blue barplots show male-specific features, red female-specific significant features. APOE: Apolipoprotein E, ethnic category: 1 = Hispanic or latino, 2 = non-hispanic, 3 = unknown [N=1], CDR: clinical dementia rating, DEMOG: patient demographic information, BA: Brodman area. Significant positive scores deviation from 0 indicate an increase in the predicted probability for AD and vice versa, the larger the magnitude (absolute value) the stronger the influence.

**Figure 3:**
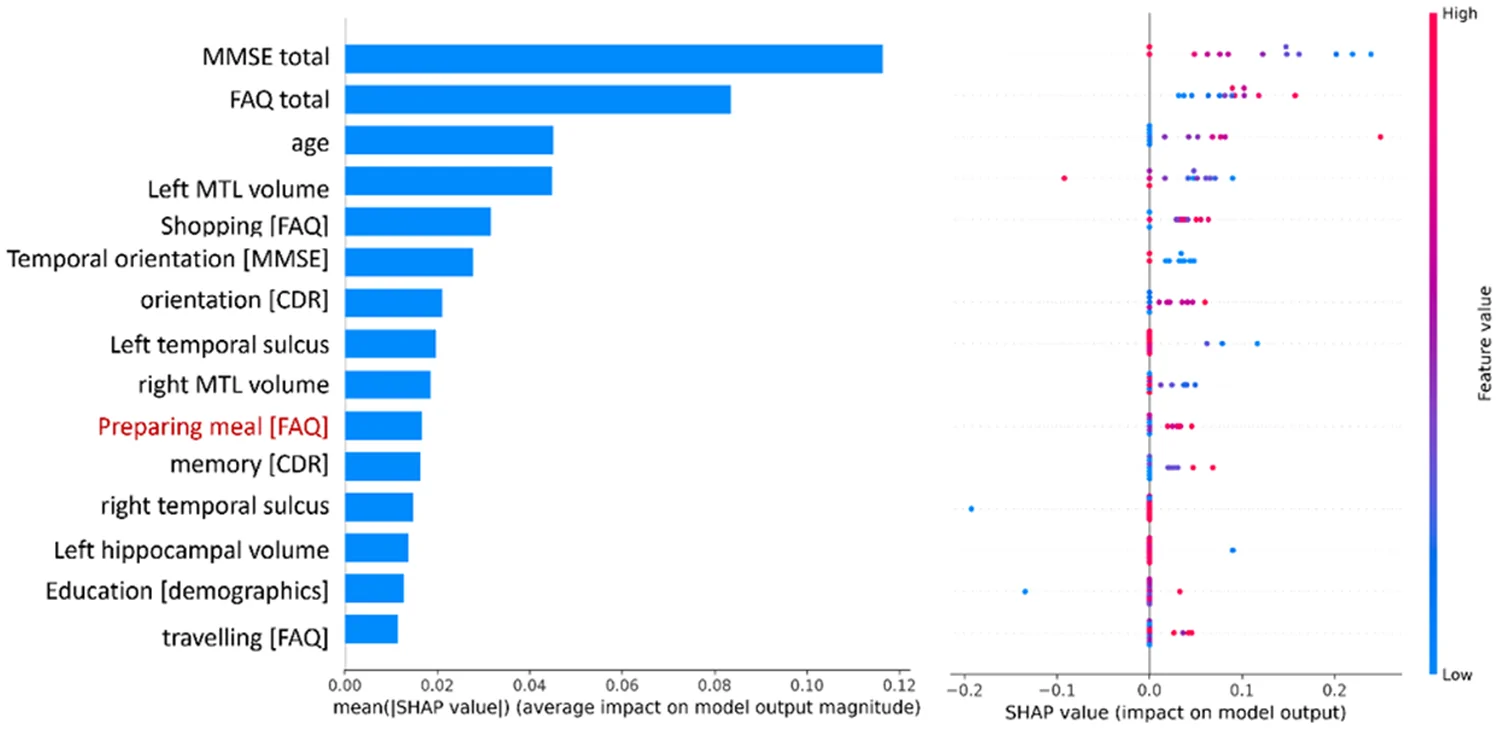
Results from XAI of Adversarial Debiasing Model: important features after debiasing. In red letters, features are highlighted, which are associated with gender gap. CDR: Clinical Dementia Rating, MMSE: Mini Mental State Exam, FAQ: Functional Activity Questionnaire

**Table 1.** Summary gender bias in MCI and AD

variable	bias	comment
verbal memory	>	preserved verbal memory masks beginning dementia in women
hippocampal atrophy	>	stronger (undetected) atrophy in women
care giving duties	>	can lead to chronic stress and depression
co-morbid depression	>	masks cognitive decline in women
marital status	<	marriage plays in favor of an early diagnosis in men
working status	<	work keeps active (men are more often the breadwinners of the family)
APOE ε4/ε4	>	higher risk in women

**Note**. APOE: Apolipoprotein E

**Table 2:** Sample demographics

Sex	MCI_women_	MCI_men_	AD_women_	AD_men_	Statstics		
	269	331	68	89	*Χ*^2^ = 0.1, p = .733		
					F_diagnosi_	F_isex_	F_interaction_
Age [yrs]	71.9 ± 8.1	74.7 ± 7.7	74.4 ± 8.6	75.5 ± 9.0	4.7, p=.031	16.5, p < .001	1.3, p = .260
CDR-SB	1.4 ± 0.9	1.5 ± 1.0	4.2 ± 2.0	4.2 ± 1.8	650.0, p < .001	0.2, p = .682	0.1, p = .751

**Note.** MCI: mild cognitive impairment, AD: Alzheimer’s Disease, CDR-SB: Clinical Dementia Rating- Sum of Boxes

**Table 3:** Results of original models and debiased models

ML	Mitigation algorithm	Original model						Debiasied model						
		bA	DI	AOD	SPD	Theil	EOD	bA	DI	AOD	SPD	Theil	EOD	N_corr_
Pre- /post-processing														
SVC	Reweighing	85.54	0.930	−0.079	−0.027	0.061	−0.129	85.54	0.935	−0.079	−0.027	0.061	−0.129	0
	Disparate Impact Remover							88.29	1.085	−0.047	0.024	0.058	−0.131	24
	Reject Option Classification							86.03	1.043	−0.041	0.016	0.061	−0.098	5
XGB	Reweighing	87.09	1.220	−0.047	0.025	0.066	−0.134	87.12	1.127	−0.034	0.028	0.066	−0.102	1
	Disparate Impact Remover							88.99	1.099	−0.048	0.024	0.056	−0.132	5
	Reject Option Classification							87.09	1.122	−0.047	0.025	0.066	−0.134	0
RF	Reweighing	88.32	0.949	−0.096	−0.017	0.056	−0.194	88.57	0.969	−0.091	−0.010	0.055	−0.194	1
	Disparate Impact Remover							89.34	0.986	−0.088	−0.004	0.052	−0.194	5
	Reject Option Classification							88.64	1.131	−0.022	0.046	0.050	−0.097	3
LR	Reweighing	85.16	0.997	−0.041	−0.017	0.071	−0.036	85.96	0.974	−0.025	−0.009	0.067	−0.004	1
	Disparate Impact Remover							**88.08**	**1.029**	**−0.024**	**0.010**	**0.055**	**−0.003**	**8**
	Reject Option Classification							86.19	1.090	0.009	0.027	0.067	0.027	5
In-processing														
	Adversarial Debiasing							**88.91**	**0.997**	**−0.076**	**−0.001**	**0.056**	**−0.165**
	Prejudice Remover							87.19	0.911	−0.080	−0.026	0.064	−0.132

**Note. Note.** SVC: Support Vector Classifier, XGB: EXtreme Gradient Boost, RF: Random Forest, LR: Logistic Regression, bA: balanced accuracy, DI: disparate impact, 1 indicates no disparity, < 1, higher likelihood of a positive outcome for the unprivileged group, > 1, higher likelihood for the privileged group; AOD: Average Odds Difference, SPD: Statistical Parity Difference, Theil: Theil Index, EOD: Equal Odds Difference, the closer to zero the fairer, N_cor_: Number of corrected patients
